# Bibliography

**Published:** 1903-07

**Authors:** 


### Bibliography.

THE FILLING OF TEETH WITH PORCELAIN (JENKINS'S SYSTEM). A Text-Book for Dentists and Students. With one hundred and ten Illustrations. By Walter Wolfgang Bruck, D.D.S., Instructor in the Dental Institute of the Royal University of Breslau. Translated from the German by Charles W. Jenkins, D.D.S., Consolidated Dental Manufacturing Company, New York.

This book of sixty-eight pages, quite profusely illustrated, will serve as an excellent guide in inlay work especially for those who purpose following the Jenkins system.

The author says, "The present work is intended to afford to the beginner, who is unacquainted with this method of filling, a knowledge of it in all its scientific bearings, and to be a faithful guide and adviser during his practical study of it." That this has been faithfuly carried out both in text and illustrations must be conceded, and the beginner in porcelain work upon this method, will find his difficulties very much lessened by carefully following the detailed directions as given by the author.

The fugitive productions by Dr. Jenkins and others have made the system very familiar to all dental operators the world over, but the practical man will frequently be at a loss when it comes to preparing an inlay, and will search probably in vain for the published paper giving instructions. The author has therefore performed an undoubted service in placing this in a permanent form most conveniently adapted to the daily needs of the practitioner. The book can, therefore, be warmly recommended as an authority upon the special system it advocates.

Success in Dental Practice. By C. N. Johnson, M.A., L.D.S., D.D.S., Professor of Operative Dentistry in the Chicago College of Dental Surgery, etc. J. B. Lippincott Company, Philadelphia and London, 1903.

The author of this timely production is a very busy man as practitioner and editor of a leading journal, yet he finds time to give dental readers the product of his active mind in permanent form. His "Principles and Practice of filling Teeth" has passed the stage of criticism and has been acepted as an authority wherever dentistry is known and practised.

The present book is the result of a matured experience in the establishment of a practice in dentistry. It is mainly intended for the young graduate, but the older practitioner may well secure from it valuable hints in the conduct of practice. Most professional men are seriously lacking in experience of business methods. They pass from school or college into the professional schools, and when graduated from the latter they are landed in a maze of inexperience. This oftentimes leads into a wreck of hope. The author says of this phase of professional life, "Commercialism is a serious menace to any profession, but there is a vast distinction between an offensive commercialism and that methodical conduct of affairs which results in a successful practice and a financial balance on the right side of the ledger at the end of each year." This is so true that it requires no argument to enforce it, and it is mainly through a lack of this "methodical conduct of affairs" that many dentists have passed beyond the meridian of life to find themselves in abject poverty when age has unfitted them for active work.

The author has performed for the profession of dentistry a signal service in pointing out to the younger generation a way to prevent this difficulty, with ordinary care at the outset of business life.

In order that our readers may have a general idea of the scope of this book, the following detailed account of the contents is given. The first chapter concerns itself with "The Arrangement of an Office," followed by "Winning Patronage, Location, Extending Acquaintance, Managing Patients, Records and Book-keeping, Appointments and Sittings, Giving Credit, Collecting Accounts, Paying Bills, Fees, Employing an Assistant, Economy in Purchase and in avoiding Waste, Bank Account and Investments, Professional Relationship and Citizenship." It will thus be seen that the author has covered the most important matters connected with practice.

While every page bristles with good suggestions, the chapters devoted to business seem, to the writer, to be most important for young men to consider, especially in keeping proper books and complete records of operations. While most practitioners have their own systems, to which they are more or less wedded, the suggestions of the author will be of value, but for simplicity and effectiveness these must commend themselves to the beginner.

The struggles of the young practitioner in beginning practice without business training have been often observed, but, beyond expressions of sympathy, no practical plans have been devised to lessen this and make his path less burdensome. The time should not be far away when a series of lectures should be given in all dental colleges upon this subject, and then, with this production as a text-book, the undergraduate would, in some degree, be prepared to face an exacting business world. Until this is done this book should be recommended to all graduating classes to be studied by them and adopted as a guide to professional success.

TRANSACTIONS OF THE AMERICAN RÖNTGEN RAY SOCIETY. Third Annual Meeting, Chicago, Ill., December 10 and 11, 1902. Louisville, 1903.

If there be needed any evidence of the rapidity with which the world of science moves, it is made evident by this publication. It seems but the other day, December, 1895, that the scientific mind of the world was startled by the announcement of Röntgen of what he called the X-ray and the results obtained. In the brief period since that day it has become a most important aid in professional work, and it is difficult to realize that less than eight years have elapsed since its discovery, yet here is a society, with a membership of over two hundred, actively engaged in this work and sending out its proceedings, covering one hundred and seventy-eight pages, containing valuable and excellent illustrations.

What may be the ultimate results of the Röntgen ray no one may tell. The few years have seen it applied to so many conditions that the student of the subject hesitates to express an opinion as to its posibilities. The present pamphlet, in some degree, outlines this and gives the present status of this interesting branch of scientific work.

The papers presented at this meeting were mainly devoted to treatment of malignant growths. The therapeutic value of the rays in this direction seems to have been demonstrated, in many cases effecting a cure and in all relieving the pain and other unpleasant symptoms. While there is much to be learned in this direction, there has been much gained in this important branch of therapeutics.

In a brief period the literature of the X-ray has grown to marked proportions, and yet before the ink is dry on the press new uses are found for it, necessitating new publications. This makes society work, with its publications, an important adjunct of progress in this direction.

